# Bacterial Contamination of Syringes and Fluids in Diagnostic and Interventional Neuroangiography

**DOI:** 10.3390/tomography10050053

**Published:** 2024-05-09

**Authors:** Martin Wiesmann, Sophia Honecker, Claudia Fleu, Christiane Franz, Manuela Schmiech, Hani Ridwan, Franziska Bürkle, Omid Nikoubashman, Sebastian Lemmen

**Affiliations:** 1 Department of Diagnostic and Interventional Neuroradiology, Medical Faculty, RWTH Aachen University Hospital, 52076 Aachen, Germany; sophia.honecker@rwth-aachen.de (S.H.); claudia.fleu@web.de (C.F.); chfranz@ukaachen.de (C.F.); hridwan@ukaachen.de (H.R.); fbuerkle@ukaachen.de (F.B.); onikoubashman@ukaachen.de (O.N.); 2 Division of Infection Control and Infectious Diseases, Medical Faculty, RWTH Aachen University Hospital, 52076 Aachen, Germany; mschmiech@ukaachen.de; 3 Institut für Krankenhaushygiene und klinische Infektiologie, Colynshofstrasse 57, 52074 Aachen, Germany; info@institut-krankenhaushygiene.de

**Keywords:** angiography, bacteremia, contamination, intervention

## Abstract

(1) Background: Bacterial contamination has been shown to occur during angiographies, although data on its frequency and relevance are sparse. Our aim was to evaluate the incidence of bacterial contamination of syringes used under sterile conditions during neuroangiographies. We sought to differentiate between contamination of the outside of the syringes and the inside and to detect the frequency, extent and germ spectrum of bacterial contamination. (2) Methods: We prospectively collected 600 samples from 100 neuroangiographies. Per angiography, fluid samples from the three routinely used syringes as well as the syringes themselves were analyzed. We analyzed the frequency and extent of contamination and determined the germ spectrum. (3) Results: The majority of samples (56.9%) were contaminated. There was no angiography that showed no contamination (0%). The outer surfaces of the syringes were contaminated significantly more frequently and to a higher extent than the inner surfaces. Both the frequency and extent of contamination of the samples increased with longer duration of angiographic procedures. Most of the bacterial species were environmental or skin germs (87.7%). (4) Conclusions: Bacterial contamination is a frequent finding during neuroangiographies, although its clinical significance is believed to be small. Bacterial contamination increases with longer duration of angiographic procedures.

## 1. Introduction

Bacteremia is proven to frequently occur after various medical procedures, such as urethral catheterization or dental procedures [1]. Bacteremia has been reported to occur in 4–8 % of angiographic procedures and is typically asymptomatic [2,3,4]. In most immune-competent patients without valvular heart disease, this does not have clinical consequences. Therefore, according to the American Heart Association guidelines for the prevention of infective endocarditis, revised in 2007 [5], and according to an adaptation by the Society of Interventional Radiology (SIR) from 2010 [6], arterial angiographic procedures are not considered to constitute a significant risk, and periprocedural antibiotics should not be given, even in patients with heart valve replacements. Nevertheless, the true incidence of bacteremia during neuroangiography is not known. Moreover, it is not known how much airborne transmission or direct contact with the patient or angio personnel contribute to bacterial contamination. In a recent study, 25.3% of all fluid samples used in diagnostic and interventional neuroangiographies were contaminated with bacteria [7]. We therefore performed an observational study to determine the rates of bacterial contamination during typical diagnostic neuroangiographies and interventional procedures. We sought to differentiate between bacterial contamination of fluids and syringes used for angiography, and we performed a detailed analysis of bacteria types.

## 2. Materials and Methods

SAMPLE COLLECTION: We prospectively collected samples during 100 neuroradiological catheter angiographies, which comprised 76 diagnostic neuroangiographies and 24 arterial neuroendovascular procedures. None of our patients had a history of ongoing bacterial infection or were under antimicrobial treatment. Angiographies or interventional procedures were performed between January and May 2021 by a team of 5 neuroradiologists. All examinations were performed in a dedicated neuroangiography suite (hygienic class Ib for procedures that do not mandate laminar air flow; DIN 1946-4). Patients’ groins were meticulously prepared for antisepsis according to the internal guidelines of our institution. Operators performed thorough disinfection of both hands up to the wrist with alcohol-based disinfectants, dressed in a sterile one-way surgical gown, and wore a cap and a face mask at all times. All procedures were performed in accordance with the recommendations of the Institute of Hygiene of our institution and with the applicable national guidelines as published by the Working Group ‘Hospital & Practice Hygiene’ of the AWMF [8]. In a standardized approach, we examined neuroangiographic fluids (sterile NaCl 0.9% solution, Fresenius, Bad Homburg, Germany) and the three angiographic 10 mL syringes (B. Braun, Melsungen, Germany) routinely used for angiographies in our department. At the end of each angiography, the three syringes were each drawn up five times with sterile NaCl 0.9% solution and the liquid was injected into one sterile container per syringe. These samples were taken to capture possible contamination of the inner surface of the syringes. The syringes themselves were then dropped into three color-coded sterile containers so that each syringe could be assigned to its rinsing liquid. These samples were taken to capture additional contamination of the outer surfaces of the syringes. Accordingly, 6 samples were collected per angiography (3 fluid samples and 3 syringes).

MICROBIOLOGICAL LABORATORY: Sample processing was performed under a laminar flow hood (Fisher Scientific, Schwerte, Germany) according to a routine hospital hygiene protocol specifically designed for the assessment of bacterial contamination in sterile fluids. The fluid samples could be used directly for sterile filtration. A total of 500 mL sterile NaCl 0.9% solution was added to each syringe and the syringes were incubated for 20 min on a shaker (Heidolph Instruments, Schwabach, Germany). For sterile filtration, an aspiration system was loaded with sterile filters (0.2 µm, Sartorius, Goettingen, Germany) and funnels (Sartorius, Goettingen, Germany) and all samples were filtered separately. One sterile filter per fluid sample was then transferred to a microbiological culture medium (TSABA Agar, Thermo Fisher Diagnostics, Wesel, Germany). Incubation was performed for 48 h in an incubator (T = 37 °C). After incubation, colonies were counted and assessed macroscopically. When necessary, individual isolates were created and incubated for an additional 24 h. Microscopic assessment was performed by classical Gram staining. Subsequently species identification was performed using Matrix-assisted laser desorption ionization–time of flight mass spectrometry (MALDI-ToF MS) (Bruker Corporation, Billerica, MA, USA).

STATISTICAL ANALYSES: Statistical analysis was performed with SPSS 28 (IBM Statistics, Chicago, IL, USA). After testing for normal data distribution with the Kolmogorow tests, *t*-tests were applied. If data did not meet the requirements of normal distribution, chi-square tests or Mann–Whitney U tests were used as applicable. The Pearson correlation coefficient was calculated to investigate the relationship between variables. Results are reported as mean ± standard deviation, or median with inter-quartile range (IQR) if not normally distributed. All tests were two-sided. A *p* value <0.05 was considered statistically significant.

## 3. Results

A total of 600 samples were taken. One sample was contaminated during sample processing and had to be excluded. Accordingly, we included 599 samples in our analysis. The 100 angiographies consisted of 76 diagnostic angiographies and 24 interventional angiographic procedures. The number of colony-forming units (CFUs) and examination duration were not normally distributed (*p* < 0.05). The median examination time for all angiographies was 1.38 h (IQR: 1.00 h; mean 1.81 ± 1.25 h). With a median duration of 1.25 h (IQR: 0.75 h; mean 1.30 ± 0.53 h), diagnostic angiographies were significantly shorter than interventional procedures, whose median duration was 3.50 h (IQR: 2.13 h; mean 3.43 ± 1.50 h) (*p* < 0.001).

### 3.1. Frequency of Contamination

Of the 599 samples evaluated, a total of 341 were contaminated (56.9%). There was no angiography that showed no contamination (0%). The outer surfaces of the syringes were significantly more frequently contaminated than the inner surfaces (*p* < 0.001, see Table 1). Contamination was found on the outside of syringes in 272 of 299 samples (91.0%). The insides of syringes were contaminated in 69 out of 300 samples (23.0%).

### 3.2. Extent of Contamination

The extent of contamination was determined based on the number of colony-forming units (CFUs). A total of 4822 CFUs were counted on the 341 contaminated samples, which corresponds to an average of 14.1 ± 41.5 CFUs per contaminated sample (median 4, IQR 8). There was significantly more contamination on the outside of the syringes than on the inside (*p* < 0.001). A total of 4682 CFUs were found on the 272 contaminated samples from the outside of the syringe (mean 17.2 ± 45.9 CFUs; median 6; IQR 10), while a total of only 140 CFUs were detectable on the 69 contaminated samples from the inside of the syringe (mean 2.0 ± 2.0 CFU; median 1, IQR 1).

### 3.3. Qualitative Analysis of Bacterial Spectrum

Qualitative analysis revealed a bacterial spectrum of 28 different species (see Table 2). The bacterial spectrum was broader on the outside of the syringes. When a sample was contaminated, an average of 3.14 ± 0.74 different microbial species were found on the outside, but only 1.35 ± 0.74 different microbial species on the inside of the syringes (*p* < 0.001). In the majority of samples from the outside of the syringes, filters were polymicrobially contaminated (73.6%) or monomicrobially contaminated (18.0%), while samples from the inside of the syringes were sterile in the majority of cases (77.3%). In contaminated samples from the inside of syringes, a single type of microbe was found in 18.0% of cases, and only 4.7% of samples were polymicrobially contaminated.

Most of the bacterial species were environmental or skin germs (87.7%). However, four groups of potentially pathogenic germs were also found: (1) *Staphylococcus aureus* and *Staphylococcus lugdunensis*; (2) *Pseudomonas* spp.; (3) *Acinetobacter* spp.; (4) Other Gram-negative germs. Environmental or skin germs were detected in all angiographies (100%). Other Gram-negative germs were found in 54%; *Staphylococcus aureus* and *Staphylococcus lugdunensis* in 15%; *Pseudomonas* spp. in 6%; and *Acinetobacter* spp. in 5% angiographies.

### 3.4. Factors Influencing the Contamination of the Samples

#### 3.4.1. Type of Angiography

Whether the angiography was a diagnostic angiography or an interventional procedure had no significant influence on the frequency of contamination or the number of CFUs found (*p* > 0.05).

#### 3.4.2. Duration of the Angiography

The longer the duration of the examination, the more frequently the samples were contaminated (*p* = 0.007, see Table 3). The contaminated samples had a mean examination duration of 1.88 ± 1.25 h (median 1.50 h; IQR 1.25 h), while the uncontaminated samples had a mean examination duration of 1.71 ± 1.24 h (median 1.25 h; IQR 0.75 h).

When samples from the inside and outside of syringes were examined separately, the contaminated samples from the outside of syringes had a highly significant longer examination time (*p* < 0.001) of 1.88 ± 1.28 h (median 1.50 h; IQR 1.25 h) compared to 1.08 ± 0.47 h (median 1.00 h; IQR 0.55 h) for uncontaminated samples. For contaminated samples from the inside of syringes, the mean examination time of 1.89 ± 1.14 h (median 1.75 h; IQR 1.00 h) was also longer than that for uncontaminated samples at 1.78 ± 1.28 h (median 1.25 h; IQR 1.00 h). However, this difference was only significant in a one-sided analysis (*p* = 0.037), but not in a two-sided analysis (*p* = 0.073).

The duration of the examination also had a significant influence on the number of CFUs found. The number of CFUs increased with longer examination duration. The correlation found (r = 0.346) was highly statistically significant (*p* < 0.001). The correlation between examination duration and number of CFUs found was significant for both samples from the inside of syringes (correlation coefficient r = 0.243; *p* < 0.015) and for samples from the inside of syringes (correlation coefficient r = 0.243; *p* < 0.015).

## 4. Discussion

The main finding of our study is that at the end of diagnostic neuroangiographies or endovascular neurointerventional procedures, the bacterial contamination of syringes, and thus of the neuroangiographic fluids which come in contact with the syringes, was detected in 100% of angiographies, even though the angiographies had been performed under routine sterile conditions.

Bacteremia has been reported to occur in 4–8% of angiographic procedures but is typically asymptomatic [2,3,4]. After dental extraction, bacteremia has even been detected in 100% of cases [1]. Usually, this does not have clinical consequences, as long as patients are immune-competent. Nevertheless, bacterial contamination is a matter of potential concern in clinical medicine. Consequently, traditional hygienic standards need to be constantly re-evaluated in hospital routine. With the increasing number and complexity of neuroendovascular procedures, this also holds true for the field of neuroradiology.

Standard angio suites do not meet operating room hygienic standards concerning air flow and patient/personnel access regulations. Infective agents are expected to be present in the air. Thus, sterile instruments or liquids may be contaminated during the procedure. The injection of such liquids as well as the endovascular use of catheters or wires may then lead to bacteremia. Our knowledge of the sources of contamination and the efficacy of potential mechanisms of avoidance is limited. Neuroangiographic procedures have not been the focus of attention with regard to bacterial contamination and have been reported to be associated with low complication rates [9]. However, potential non-microbial contamination has been reported [10]. Tress et al. [11] described contamination in three out of seven patients who underwent angiography. In another study, the contamination of sterile liquids occurred during neuroangiographies in approximately 25% of cases [7]. The authors were not able to determine the route of contamination. However, they considered airborne transmission unlikely since intermittent coverage of the fluid bowls did not lead to a reduction in contamination.

During angiographies, contrast agents and water are injected using syringes. That is why it was the purpose of our study to determine whether syringes remain sterile during angiographies. We also sought to discern between the contamination of the inside and the outside of syringes. A certain frequency of contamination of the outside of syringes was to be expected, be it through airborne bacterial transmission or through contact with contaminated cloths or gloves. Potentially, this need not always have consequences for the patient. On the contrary, contamination of the inside of syringes, if these syringes were used for injections, would almost inevitably result in bacteremia for the patient.

We found that the majority of syringes (56.9%) were contaminated at the end of angiographies, and that there was not a single angiography which showed no contamination. As expected, the outer surfaces of the syringes were significantly more frequently contaminated than the inner surfaces. In fact, nearly all syringes were contaminated on the outside (91.0%). We had not expected, however, that a considerable portion of the syringes (23.0%) would also be contaminated on the inside. This means that it is likely that bacteremia was caused during angiography in some cases. Our data show that both the frequency and the extent of contamination increase with the duration of angiography. The relationship between longer duration of angiographies and bacterial contamination was highly significant for samples from the outer surfaces of syringes but weak for samples from the inner surfaces of syringes.

Our study cannot give definitive answers with regard to the route of bacterial transmission. Considering the high frequency of contamination we found, we would tend to agree with Kabbasch et al. [7] and hypothesize that airborne transmission does not play a major role. Instead, the repetitive contact of the syringes with the operator’s gloves seems to be a more likely explanation. This is in line with our observation that longer procedural durations lead to significantly increased contamination on the outer surfaces of syringes, since the number of times the operator’s gloves come into contact with a syringe is correlated with procedural duration. However, this needs to be confirmed in future studies.

The majority of the germ species we detected were environmental or skin germs (87.7%). However, in our samples, we also found a small portion of potentially pathogenic germs, such as Staphylococcus aureus or *Pseudomonas* spp. Nevertheless, this will most probably not have had any consequences for immune-competent patients, considering that even brushing one’s teeth has been shown to cause relevant bacteremia in 23% of cases in some series [12]. On the other hand, it is a shortcoming of our study that we did not test for actual bacteremia and the follow-up period for our patients was too short to definitely rule out any clinical effects. Consequently, we cannot estimate the true incidence of bacteremia in our patients.

Although we are convinced that angiographically induced bacteremia in the standard patient population is clinically completely irrelevant, we nevertheless believe that we should strive to reduce the frequency and extent of bacterial contamination during angiography, especially with regard to critically ill or immuno-incompetent patients. With this goal in mind, our results can serve as a starting point for future studies.

## 5. Conclusions

Bacterial contamination is a frequent finding during neuroangiographies, although its clinical significance is believed to be small. Bacterial contamination increases with longer duration of angiographic procedures. The repetitive contact of syringes with the gloves of the operator seems to be the most likely cause of contamination, although this needs to be confirmed in future studies.

## 6. Limitations

We are aware of the shortcomings of our study as this was a descriptive study only and we did not vary the angiographic conditions to test for potential effects on bacterial contamination. We also did not investigate actual bacteremia in our patients, nor did we add to clinical routine to detect delayed complications secondary to bacteremia.

## Figures and Tables

**Table 1:** Contamination of syringes.

	Outer Surfaces of Syringes	Inner Surfaces of Syringes	*t*-Test
Percentage of contaminated samples	91.0%	23.0%	*p* < 0.001
Colony-forming units (CFUs)	17.2 ± 45.9	2.0 ± 2.0	*p* < 0.001
No. of different microbial species	3.14 ± 0.74	1.35 ± 0.74	*p* < 0.001

**Table 2:** Bacterial spectrum found in the samples using MALDI-ToF MS.

Germ Species	Gram	Group
*Acinetobacter* spp.	negative	potentially pathogenic
*Aerococcus viridans*	negative	other Gram-negative germs
*Bacillus* spp.	positive	environmental or skin germs
*Brachybacterium muris*	positive	environmental or skin germs
*Brevibacillus* spp.	positive	environmental or skin germs
*Brevibacterium celere*	positive	environmental or skin germs
*Brevundimonas* spp.	negative	other Gram-negative germs
*Corynebacterium* spp.	positive	environmental or skin germs
*Dermabacter hominis*	positive	environmental or skin germs
*Dermacoccus* spp.	positive	environmental or skin germs
*Enterococcus faecalis*	positive	environmental or skin germs
*Gordonia hongkongensis*	positive	environmental or skin germs
*Kocuria* spp.	positive	environmental or skin germs
*Kytococcus* spp.	positive	environmental or skin germs
*Lactococcus lactis*	positive	environmental or skin germs
*Micrococcus* spp.	positive	environmental or skin germs
*Moraxella* spp.	negative	other Gram-negative germs
*Paenibacillus* spp.	positive	environmental or skin germs
*Pantoea* spp.	negative	other Gram-negative germs
*Paracoccus yeei*	negative	other Gram-negative germs
*Pseudarthrobacter* spp.	positive	environmental or skin germs
*Pseudoclavibacter* spp.	positive	environmental or skin germs
*Pseudomonas* spp.	negative	potentially pathogenic
*Roseomonas mucosa*	negative	environmental or skin germs
*Solibacillus silvestris*	positive	environmental or skin germs
*Sphingomonas* spp.	negative	environmental or skin germs
*Staphylococcus aureus*	positive	potentially pathogenic
*Staphylococcus lugdunensis*	positive	potentially pathogenic

**Table 3:** Effect of the duration of angiographies on microbial contamination.

	Contaminated Samples	Uncontaminated Samples	*t*-Test
Duration of angiography [h] (all samples)	1.88 ± 1.25	1.71 ± 1.24	*p* = 0.007
Duration of angiography [h] (samples from outer surfaces of syringes)	1.88 ± 1.28	1.08 ± 0.47	*p* < 0.001
Duration of angiography [h] (samples from inner surfaces of syringes)	1.89 ± 1.14	1.78 ± 1.28	*p* = 0.073 (not significant)

## Data Availability

The raw data supporting the conclusions of this article will be made available by the authors on reasonable request.
